# Evaluation of perceived depression, anxiety, stress levels and emotional eating behaviours and their predictors among adults during the COVID-19 pandemic

**DOI:** 10.1017/S1368980022002579

**Published:** 2023-03

**Authors:** Gülşah Kaner, Gamze Yurtdaş-Depboylu, Gamze Çalık, Tuba Yalçın, Tutku Nalçakan

**Affiliations:** Izmir Katip Celebi University, Faculty of Health Sciences, Departments of Nutrition and Dietetics, Balatçık Mahallesi Havaalanı Şosesi No: 33/2 Balatçık, Çiğli, Izmir 35620, Turkey

**Keywords:** COVID-19, Anxiety, Depression, Stress, Emotional eating

## Abstract

**Objective:**

This study aimed to (1) evaluate the prevalence and predictors of perceived depression, anxiety, stress (DAS) levels and emotional eating behaviours and (2) determine the correlations between DAS levels and emotional eating behaviours during the pandemic.

**Design:**

An online cross-sectional study included questions about demographic and anthropometric characteristics, dietary habits, Emotional Appetite Questionnaire (EMAQ) and Depression Anxiety Stress Scales. The snowball sampling method was used.

**Setting:**

Türkiye.

**Participants:**

The study population was 2002 adults aged ≥18 years.

**Result:**

The mean age was 27·1 ± 9·52 years (72·1 % females and 27·9 % males). The prevalence of moderate to severe DAS was reported as 27·8 %, 30·5 % and 30·7 %, respectively. Skipping meals (OR = 1·32, 95 % CI (1·14, 1·49)) was associated with depression. Weight gain (OR = 1·43, 95 % CI (1·19, 1·66); OR = 1·30, 95 % CI (1·14, 1·49); OR = 1·39, 95 % CI (1·14, 1·64)), weight loss (OR = 1·45, 95 % CI (1·20, 1·70); OR = 1·37, 95 % CI (1·11, 1·62); OR = 1·46, 95 % CI (1·20, 1·72)), exercising at least 150 min/week (OR = 0·64, 95 % CI (0·46, 0·83); OR = 0·73, 95 % CI (0·55, 0·92); OR = 0·83, 95 % CI (0·63, 1·02)), and maintaining an adequate and balanced diet (OR = 0·52, 95 % CI (0·33, 0·71); OR = 0·53, 95 % CI (0·34, 0·73); OR = 0·63, 95 % CI (-0·15, 0·35)) were associated with DAS, respectively. BMI (*r* = 0·169, *P* < 0·001), weight (*r* = 0·152, *P* < 0·001), number of snacks (*r* = 0·102, *P* = 0·011), depression (*r* = 0·060, *P* = 0·007), anxiety (*r* = 0·061, *P* = 0·006) and stress (*r* = 0·073, *P* = 0·001) levels were positively correlated with EMAQ-negative scores.

**Conclusion:**

Approximately one out of every three participants reported moderate to severe DAS levels. Emotional eating was significantly correlated with perceived DAS. The predictors obtained in the study suggest that a healthy diet and lifestyle behaviours are part of psychological well-being and emotional eating.

The coronavirus disease (COVID-19) is described as a worldwide outbreak that causes deaths and seriously threatens all humanity. The WHO announced a global public health crisis in January 2020 after the first cases of COVID-19 were discovered in China^(1,2)^. The first case was recorded on 11 March 2020, in Turkey, and spread to the entire country in a short time. Serious measures have been taken to slow the transmission of this infection in Turkey. People were not allowed to leave their houses except in emergency situations, and non-essential activities were moved online or closed to ensure social isolation^(3)^. Although these restrictions are necessary to prevent the transmission of the virus, the sudden start of quarantine, the uncertainty of the process, staying at home by avoiding social relations and the ever-increasing number of infected individuals increased the risk of mental symptoms and eating behaviours^(4,5)^. A systematic review of sixty-two studies that conducted during the pandemic from seventeen countries, including Türkiye, reported the prevalence of depression and anxiety to be 28 % and 33 %, respectively^(6)^.

Individuals may exhibit different eating behaviours to overcome anxiety, depressive mood and other negative emotions^(7)^. Stress and negative mood can cause loss of appetite for many individuals^(8)^. However, for the vast majority of individuals, stress and negative emotions can induce them to eat more^(9)^. Emotional eating is often described as overeating in response to negative moods^(10)^. Previous research has revealed that emotional eating is positively related to BMI^(5,11)^ and stress^(12)^. It was found that subjects who were overweight and experienced negative moods ate more food than normal-weight and underweight subjects^(13)^.

It is therefore worth evaluating the depression, anxiety, stress (DAS) levels and emotional eating behaviours during the pandemic. To the best of our knowledge, no research has specifically investigated the prevalence and predictors of perceived DAS levels and emotional eating behaviours during the pandemic in the Turkish adult population. In the present study, we examined factors associated with perceived DAS levels and emotional eating behaviours and examined correlations between DAS and emotional eating behaviours. Our findings could provide a framework for an understanding of DAS levels and emotional eating behaviours and their predictors during the pandemic.

## Methods

### Design and participants

**Fig. 1:**
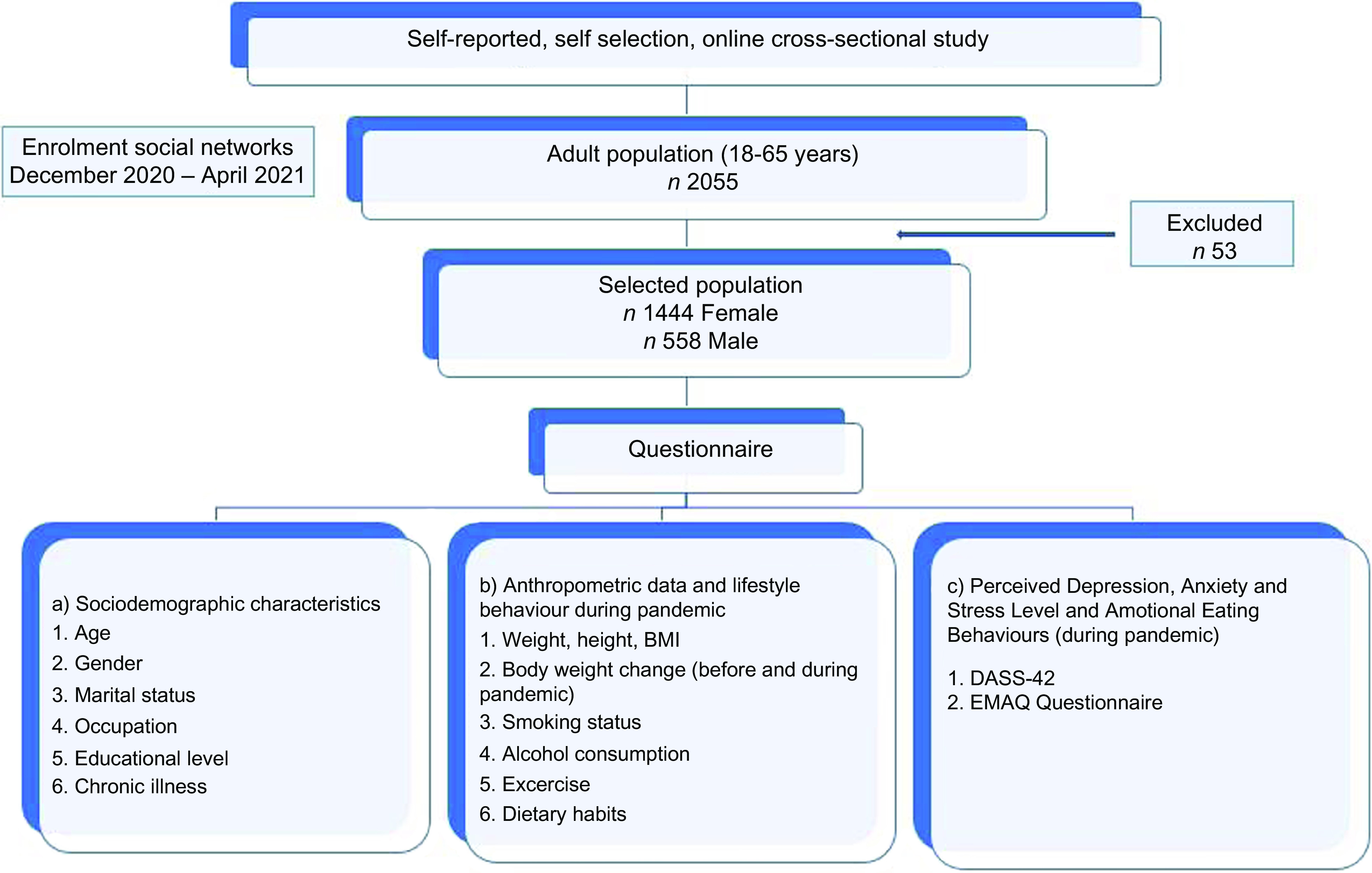
Study flow diagram. DASS, Depression, Anxiety, Stress Scale; EMAQ, Emotional Appetite Questionnaire

This self-selection online cross-sectional study was carried out among participants aged 18–65 years in Türkiye. Inclusion criteria were as follows: (1) using WhatsApp; (2) residing in Türkiye; and (3) being 18 years or older. Participants excluded from the study were those (1) infected with COVID-19 at the time of the research, (2) who had psychological and eating disorders, and (3) who did not complete the survey. Moreover, participants who reported that they were pregnant or breast-feeding during the pandemic were excluded before the analysis. Participants were asked to provide their email addresses for avoiding duplicate submissions. Duplicate submissions having the same email address were removed from the analysis. The study flow diagram is presented in Fig. 1.

### Data collection

Self-reported data collection was carried out from December 2020 to April 2021 using Google Forms web survey software. A snowball sampling technique was used for this survey. Respondents were recruited via social networks (Facebook, WhatsApp student groups and Instagram). Survey invitations were systematically shared on Facebook and Instagram at different times of day and days of the week, and others were encouraged to share the survey invitation. Necessary corrections in the questionnaire were made after receiving feedback from ten early responses. The online questionnaire form used in this study consists of six sections. The first section included questions about general features such as gender (male and female), age, marital status (married and single), occupation (unemployed, health sector, private sector, officer, student and retired) and education level (literate, primary, secondary, and high school, university, master or PhD). The second section included health information such as chronic disease status, smoking and alcohol consumption status with possible answers ‘yes’ or ‘no’. Moreover, individuals were asked to self-report about exercising at least 150 min/week during the pandemic. Response options included ‘yes’ or ‘no’. The third section included questions about dietary habits. The fourth and fifth sections included the ‘Depression, Anxiety, Stress Scale (DASS-42)’ and ‘Emotional Appetite Questionnaire (EMAQ)’, respectively. The last section included self-reported body weight and height.

### Dietary habits

The part of the questionnaire that was used in our research related to dietary habits included the following questions: Do you skip the main meal? If the respondents answered ‘yes’ to this question, we asked three more questions. ‘Do you skip breakfast/lunch/dinner?’. In the previous questions, respondents should answer ‘yes’ or ‘no’. The question about maintaining healthy dietary habits was as follows: ‘Do you think that you maintain healthy dietary habits during the COVID-19 pandemic?’ with possible answers ‘yes’ or ‘no’. This result is self-reported perceptions of participants. Respondents were asked to answer this question by taking into consideration the recommendations of the Turkish Dietary Guideline (eating a diversity of foods in each food group, consuming a diet rich in whole grains and nuts, eating plenty of fruits and vegetables, consuming healthy fats rich in unsaturated fatty acids, drinking water regularly, following intake of fats, sugar and salt)^(14)^.

### Depression, Anxiety, Stress Scale (DASS-42)

The DASS-42 is a self-report questionnaire that evaluates DAS levels. The original scale was developed in 1995 by Lovibond and Lovibond^(15)^. Akın and Çetin translated the questionnaire’s validity and reliability into Turkish in 2007^(16)^.

The DASS-42 is devised as a forty-two-item instrument consists of three subscales within fourteen items. For each subscale, there are options ranging from zero (did not apply to me at all) to three (applied to me very much). The total scores of the scale without reversed items range from zero to forty-two for each sub-dimension. The normal score ranges for DAS are 0–9, 0–7 and 0–14, respectively. Total scores above these ranges are classified into four categories: mild (range is 10–13 for depression, 8–9 for anxiety and 15–18 for stress), moderate (range is 14–20 for depression, 10–14 for anxiety and 19–25 for stress), severe (range is 21–27 for depression, 15–19 for anxiety and 26–33 for stress) and extremely severe (range is ≥ 28 for depression, ≥20 for anxiety and ≥34 for stress)^(16)^.

The DASS-42 scale has been a reliable and valid instrument for assessing perceived DAS within the Turkish population. The Turkish form’s Cronbach’s *α* internal consistency reliability coefficients were reported as 0·90 for depression, 0·92 for anxiety and 0·92 for stress^(16)^. In another study, internal consistency coefficients were 0·92 for depression, 0·86 for anxiety and 0·88 for stress^(17)^. These values point out the Turkish version of DASS-42 has acceptable internal consistency. In the present study, Cronbach’s *α* was 0·95, 0·91 and 0·95 for DAS, respectively.

### The Emotional Appetite Questionnaire

The EMAQ was developed to evaluate the relationship between eating behaviours and positive/negative emotions and situations in individuals by Nolan *et al.* in 2010^(18)^. The validity and reliability of the questionnaire were adapted to Turkish in 2014 by Demirel *et al.* This questionnaire consists of twenty-two items in a nine-point Likert type. The participants scored each item in the scale in terms of how much certain situations and emotions affect their appetite as less (1–4), the same (5) and more (6–9). Emotional eating presence is evaluated in negative/positive emotions (fourteen items) and negative/positive situations (eight items). The EMAQ-positive total score is obtained by adding up the scores of positive emotions (3, 6, 11, 12, 14) and situations (18, 20, 22), while the EMAQ-negative total score is obtained by adding the scores of negative emotions (1, 2, 4, 5, 7, 8, 9, 10, 13) and situations (15, 16, 17, 19, 21). This questionnaire does not have any cut-off points for emotional eating^(19)^.

### Anthropometric measurements

Self-reported height (cm) and current body weight (kg) were obtained at the time of the survey. BMI was calculated by dividing body weight in kilograms by the square of height in metres. Participants were classified as underweight (<18·50 kg/m^2^), normal weight (18·50–24·99 kg/m^2^), overweight (25·00–29·99 kg/m^2^) and obese (≥30·00 kg/m^2^) according to the cut-off points determined by the WHO^(20)^. Moreover, participants were asked for information on body weight change before and during the pandemic (no change, increase and decrease).

### Statistical analysis

Data were analysed with SPSS 22.0 (SPSS Inc.) statistical package program. The Kolmogorov–Smirnov test was used to determine the data’s conformity to normal distribution. Categorical data were expressed as numbers (*n*) and percentages (%), normally distributed data were shown as the mean ± sd ( 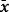 ± sd), and non-normally distributed data as median ± interquartile range. Parametric methods were used to analyse normally distributed data, while non-parametric methods were used to analyse non-normally distributed data. In the comparison of the measurement values of two independent groups, normally distributed data were analysed using the ‘independent samples *t* test’, and data that were non-normally distributed were analysed using the ‘Mann–Whitney U test’. In the comparison of the measurement values of three or more independent groups, normally distributed data were analysed with the ‘ANOVA test’ and data that were non-normally distributed were analysed with the ‘Kruskal–Wallis H test’. Ordinal logistic regression was used to evaluate association between dietary habits and lifestyle behaviours and DAS. The model was adjusted by age, marital status, gender, chronic disease status, educational level and occupation. The OR and 95 % CI were estimated for each factor. Pearson’s or Spearman’s correlation was performed to examine the correlation between DASS-42, EMAQ-negative and positive subscales. For all analyses, statistical significance was considered *P* < 0·05.

## Results

The sociodemographic features of the individuals are shown in Table 1. A total of 2055 participants filled out the survey. From this sample, participants with missing values, outliers for some questions or duplicate submissions (*n* 39), participants who reported having been infected by COVID-19 (*n* 7), pregnant (*n* 2) and participants who were less than 18 years of age (*n* 5) were excluded. The remaining 2002 participants (mean age 27·1 ± 9·52 years, 1444 female) were included in the study. The majority of the participants (*n* 1759, 87·9 %) reported that they did not use alcohol, and 71·5 % did not smoke.

**Table 1:** General characteristics of participants (*n* 2002)

		*n*	%
Age (years)	18–29	1444	72·1
	30–39	269	13·5
	40–49	211	10·5
	50–65	78	3·9
Gender	Male	558	27·9
	Female	1444	72·1
Marital status	Married	488	24·4
	Single	1514	75·6
Occupation	Unemployed	140	7·0
	Health sector	181	9·0
	Private sector	461	23·0
	Officer	331	16·5
	Student	868	43·4
	Retired	21	1·0
Education level	Literate	91	4·5
	Primary school	15	0·7
	Secondary school	32	1·6
	High school	798	39·9
	University	877	43·8
	Master or PhD	186	9·3
Chronic illness	Yes	589	29·4
	No	1413	70·6
Smoking status	Yes	571	28·5
	No	1431	71·5
Alcohol consumption	Yes	243	12·1
	No	1759	87·9

**Fig. 2:**
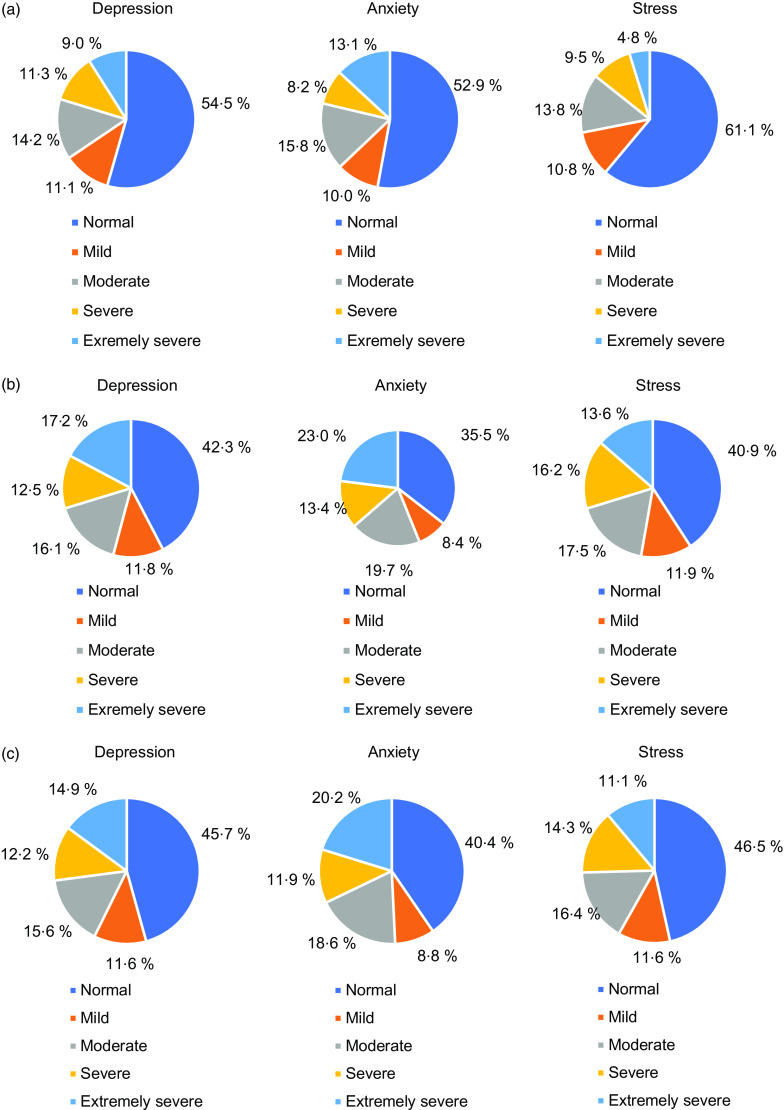
(a) Perceived depression, anxiety and stress levels of males; (b) perceived depression, anxiety and stress levels of females; (c) perceived depression, anxiety and stress levels of all participants

Figure 2 shows the prevalence of self-perceived DAS levels. According to DASS-42, the prevalence of moderate to severe DAS was reported as 27·8 %, 30·5 % and 30·7 %, respectively.

The distribution of DASS-42 and EMAQ levels according to some factors is given in Table 2. The DAS levels of females were higher than males (*P* < 0·05). The DAS levels of subjects who declared they had an adequate and balanced diet were significantly lower than those who did not think or sometimes thought that they had an adequate and balanced diet (*P* < 0·05). The DAS levels of underweight participants were higher than normal weight and overweight (*P* < 0·05). The DAS levels of participants whose body weight decreased and increased were significantly higher than participants whose body weight did not change (*P* < 0·05). The DAS levels of participants who reported exercising at least 150 min/week were lower than those who did not (*P* < 0·05). A significant difference was established in terms of DAS scores according to skipping the main meals (*P* < 0·05).

**Table 2:** Difference in DASS-42 and EMAQ subscale scores based on gender, chronic disease status, eating habits, BMI, weight change and physical activity

		Depression		*P* *	Anxiety		*P* *	Stress		*P* *	EMAQ-negative		*P* **	EMAQ-positive		*P* **
		Median	IQR		Median	IQR		Median	IQR		Mean	sd		Mean	sd	
Age (years)	18–29 (*n* 1444)	13·0	19·0	**<0·001**	11·0	13·0	**<0·001**	18·0	18·0	**<0·001**	42·6	22·66	**0·003**	33·5	15·13	**0·018**
	30–39 (*n* 269)	8·0	17·0	a > b,c,d†	8·0	13·0	a > b,c,d†	13·0	18·0	a > b,c,d†	45·6	24·42	a,b > c§	32·1	14·28	a > c§
	40–49 (*n* 211)	5·0	10·0		5·0	9·0		9·0	11·0		38·0	22·42		30·2	14·53	
	50–65 (*n* 78)	5·0	10·0		6·5	9·0		10·0	15·0		44·5	20·00		33·9	13·72	
Gender	Male (*n* 558)	8·0	15·0	**<0·001**	7·0	10·0	**<0·001**	12·0	15·0	**<0·001**	42·1	23·31	0·493	34·6	16·34	**<0·001**
	Female (*n* 1444)	12·0	18·0		11·0	14·0		18·0	19·0		42·8	23·05		32·3	14·31	
Chronic illness	Yes (*n* 589)	14·9	19·0	**<0·001**	12·0	14·0	**<0·001**	18·0	20·0	**<0·001**	44·3	24·33	**0·033**	32·4	14·65	0·264
	No (*n* 1413)	10·0	17·0		9·0	12·0		15·0	17·0		41·9	22·17		33·2	15·05	
BMI (kg/m^2^)	Underweight (*n* 197)^a^	13·0	19·0	**0·011**	12·0	15·0	**<0·001**	19·0	18·0	**<0·001**	37·1	21·53	**<0·001**	35·1	15·09	0·195
	Normal (*n* 1203)^b^	11·0	17·0	a > b,c,d†	10·0	12·0	a > b,c,d†	16·0	17·0	a > b,c,d†	40·9	21·33	a < c, d‡	32·6	14·76	
	Overweight (*n* 446)^c^	10·0	19·0		9·0	13·0		14·0	18·0		47·7	25·09	b < c,d‡	33·0	14·99	
	Obese (*n* 156)^d^	9·0	20·0		8·0	16·0		12·0	20·0		48·4	25·43		32·6	15·80	
Weight change (kg)	Maintained weight (*n* 340)^a^	7·5	15·0	**<0·001**	7·0	11·0	**<0·001**	12·0	16·0	**<0·001**	39·9	20·55	**0·001**	33·7	15·08	**0·039**
	Gained weight (*n* 1020)^b^	11·0	18·0	b,c>a†	10·0	13·0	b,c>a†	16·0	18·0	b,c>a†	44·4	23·87	b > c,a‡	31·8	14·76	a > b§
	Lost weight (*n* 642)^c^	13·0	19·0		10·0	14·0		17·0	18·0		41·2	22·12		32·7	14·71	
Exercising for at least 150 min/week	Yes (*n* 553)	9·0	16·0	**<0·001**	9·0	11·0	**0·001**	14·0	17·0	**0·015**	41·8	22·47	0·338	32·3	15·03	0·197
	No (*n* 1449)	11·0	18·0		10·0	13·0		16·0	18·0		42·9	22·99		33·2	14·89	
Maintain an adequate and balanced diet	Yes (*n* 1014)^a^	7·0	14·0	**<0·001**	7·0	10·0	**<0·001**	12·0	15·0	**<0·001**	41·7	22·03	0·140	33·0	14·77	0·397
	No (*n* 361)^b^	18·0	20·0	a < b,c†	14·0	15·0	a < b,c†	22·0	18·0	a < b,c†	44·1	24·84		32·1	15·14	
	Sometimes (*n* 627)^c^	14·0	17·0		12·0	13·0		18·0	17·0		43·3	22·91		33·4	15·07	
Skipping the main meal	Yes (*n* 1226)	13·0	18·0	**0·001**	11·0	13·0	**0·009**	17·0	18·0	**0·003**	42·7	23·25	0·886	32·6	14·85	0·219
	No (*n* 776)	8·0	15·0		8·0	12·0		14·0	18·0		42·5	22·20		33·5	15·06	
Skipping breakfast	Yes (*n* 465)	13·0	19·0	**<0·001**	11·0	14·0	**0·001**	17·0	18·0	**0·001**	43·4	24·24	0·802	33·1	14·78	0·424
	No (*n* 1537)	10·0	18·0		9·0	13·0		15·0	17·0		42·4	22·41		32·9	14·99	
Skipping lunch	Yes (*n* 785)	12·0	18·0	**0·001**	11·0	13·0	**0·004**	17·0	19·0	**0·001**	41·9	22·94	0·289	32·4	14·76	0·182
	No (*n* 1217)	10·0	18·0		9·0	13·0		15·0	18·0		43·0	22·78		33·3	15·04	
Skipping dinner	Yes (*n* 85)	17·0	21·0	**0·013**	13·0	17·0	**0·007**	19·0	20·0	**0·043**	42·1	25·40	0·842	31·4	14·53	0·318
	No (*n* 1916)	11·0	17·0		10·0	13·0		16·0	18·0		42·6	22·73		33·0	14·95	

DASS, Depression, Anxiety, Stress Scale; EMAQ, Emotional Appetite Questionnaire.

* Kruskal–Wallis *H* test.

† Mann–Whitney *U* test.

‡ Tukey’s test.

§ Tamhane’s T2.

** One-way ANOVA.

The bold values indicate significance at *P* < 0·05.

The mean EMAQ-positive score of the males was significantly higher than that of the females (*P* < 0·05). The mean EMAQ-negative score was significantly higher in overweight and obese subjects compared with normal-weight and underweight subjects (*P* < 0·05). Participants who declared increased body weight had a higher mean EMAQ-negative score than participants who declared decreased and maintained body weight (*P* < 0·05) (Table 2).

The ordinal logistic regression analysis of the factors associated with DAS symptoms are shown in Table 3. After adjustment for age, educational level, gender, marital status, occupation and chronic disease status, participants who lost/gained body weight during COVID-19 had an increased likelihood of DAS compared with participants who maintained body weight (*P* < 0·05). Participants who reported that they exercised at least 150 min/week had a decreased likelihood of depression (OR = 0·64, 95 % CI (0·46, 0·83)), anxiety (OR = 0·73, 95 % CI (0·55, 0·92)) and stress (OR = 0·83, 95 % CI (0·63, 1·02)) symptoms. Participants who reported skipping the main meal were more likely to have higher depression symptoms (OR = 1·32, 95 % CI (1·14, 1·49)), whereas skipping the main meal was not associated with anxiety and stress symptoms. Participants who declared that they have not an adequate and balanced diet were more likely to have higher depression (OR = 1·68, 95 % CI (1·45, 1·91)), anxiety (OR = 1·44, 95 % CI (1·21, 1·68)) and stress (OR = 1·42, 95 % CI (1·18, 1·65)) symptoms.

**Table 3:** The ordinal logistic regression analysis of the factors associated with depression, anxiety and stress symptoms

Characteristic		Depression					Anxiety					Stress				
		Estimate	SE	OR	95 % CI	*P*-value	Estimate	SE	OR	95 % CI	*P*-value	Estimate	SE	OR	95 % CI	*P*-value
Weight change	Gained weight	0·361	0·122	1·43	1·19, 1·66	**0·002**	0·264	0·121	1·30	1·06, 1·53	**0·029**	0·333	0·126	1·39	1·14, 1·64	**0·008**
	Lost weight	0·375	0·130	1·45	1·20, 1·70	**0·003**	0·315	0·130	1·37	1·11, 1·62	**0·015**	0·381	0·134	1·46	1·20, 1·72	**0·005**
	Maintained weight*															
Exercising for at least 150 min a week	Yes	−0·431	0·095	0·64	0·46, 0·83	**<0·001**	−0·304	0·095	0·73	0·55, 0·92	**0·001**	−0·185	0·098	0·83	0·63, 1·02	**0·006**
	No*															
Maintain an adequate and balanced diet	Yes	−0·639	0·097	0·52	0·33, 0·71	**<0·001**	−0·620	0·098	0·53	0·34, 0·73	**<0·001**	−0·461	0·100	0·63	-0·15, 0·35	**<0·001**
	No	0·522	0·119	1·68	1·45, 1·91	**<0·001**	0·370	0·120	1·44	1·21, 1·68	**0·002**	0·352	0·121	1·42	1·18, 1·65	**0·004**
	Sometimes*															
Skipping the main meal	Yes	0·279	0·089	1·32	1·14, 1·49	**0·002**	0·119	0·096	1·12	0·93, 1·31	0·219	0·189	0·098	1·20	1·01, 1·40	0·054
	No*															
Skipping breakfast	Yes	0·039	0·110	1·00	0·82, 1·25	0·724	0·037	0·110	1·03	0·82, 1·25	0·734	−0·011	0·111	0·98	0·77, 1·20	0·923
	No*															

* Reference group.

Adjusted for age, education level, gender, marital status, occupation and chronic disease status.

Significant values are shown in bold (*P* < 0·05).

There was a significant positive correlation between EMAQ-negative score and depression (*r* = 0·060, *P* = 0·007), anxiety (*r* = 0·061, *P* = 0·006) and stress levels (*r* = 0·073, *P* = 0·001). A significant positive correlation was found between EMAQ-negative score and body weight (*r* = 0·152, *P* < 0·001), BMI (*r* = 0·169, *P* < 0·001) and number of snacks (*r* = 0·102, *P* = 0·011). There was a significant positive correlation between the EMAQ-positive score and the number of snacks (*r* = 0·057, *P* = 0·011) (Table 4). However, these correlation coefficients were weak.

**Table 4:** Correlation of DASS-42, EMAQ-negative and positive subscales

	EMAQ-negative		EMAQ-positive	
	*r*	*P*	*r*	*P*
Body weight (kg)	0·152	**<0·00**1*	0·007	0·743*
BMI (kg/m²)	0·169	**<0·001** *	−0·024	0·282*
Number of meals	0·003	0·889†	−0·005	0·861†
Number of snacks	0·102	**0·011** †	0·057	**0·011** †
DASS-depression	0·060	**0·007** *	0·007	0·759*
DASS-anxiety	0·061	**0·006** *	0·001	0·955*
DASS-stress	0·073	**0·001** *	0·038	0·089*

DASS, Depression, Anxiety, Stress Scale; EMAQ, Emotional Appetite Questionnaire.

* Spearman’s correlation test.

† Pearson’s correlation test.

Significant values are shown in bold (*P* < 0·05).

## Discussion

This cross-sectional survey was conducted to evaluate the prevalence and predictors of perceived DAS levels and emotional eating behaviours among Turkish adults during the outbreak. The following are the main objectives of this study: it was determined that approximately one out of every three individuals perceived moderate to severe DAS during the pandemic. Weight gain, weight loss, exercising at least 150 min/week, maintaining an adequate and balanced diet were factors independently correlated with DAS. BMI, body weight, number of snacks and DAS levels were positively correlated with EMAQ-negative scores.

Previous reports show that the pandemic may have significant effects on psychological distress^(21,22)^. In the study carried out to determine the immediate psychological response to the pandemic in China, it was shown that moderate to severe DAS levels were 16·5 %, 28·8 % and 8·1 %, respectively^(21)^. In the study conducted to investigate the relationship between DAS levels, and emotional eating behaviours in Türkiye, it was shown that 34·8 % of the participants perceived moderate to severe depression, 23·9 % perceived moderate to severe anxiety and 32·1 % perceived moderate to severe stress^(23)^. Similar to the study by Kalkan Uğurlu *et al.*, the findings from the present study show that 27·8 % of participants indicated moderate to severe depressive symptoms, 30·5 % of participants indicated moderate to severe anxiety symptoms and 30·7 % indicated moderate to severe stress levels. In our study, the respondents’ levels of DAS were higher in the study by Wang *et al*.^(21)^ Since the data collection started about 8 months after the first COVID-19 case appeared in Türkiye, we thought that this may have been due to the late psychological responses to the pandemic.

Our results showed that the female gender was correlated with elevated DAS levels. This result is consistent with the UN Policy Brief report^(24)^. It was pointed out that females’ knowledge score about COVID-19 was higher than males and paid more attention to suggestions such as staying away from crowded places and wearing a mask^(25)^. A UN Policy Brief report indicates that women have experienced increased domestic violence during the pandemic. Moreover, it was reported that caregiving duty (homeschooling and taking care of elder relatives) of women increased during the pandemic. Additionally, it was indicated that pandemic can lead to negative moods related to economic crisis, fear of losing family members or threat of unemployment^(26)^.

COVID-19 required to isolate citizens in their homes for weeks. Recent study has warned of a possible increase in individuals’ psychological problems^(27)^. The stressful environment caused by COVID-19 home confinement led to an increase or decrease in individuals’ weight^(28,29)^. COVID-19 stressors may have triggered depressive symptoms and induced maladaptive eating behaviours such as overeating or restrictive eating. It also causes major weight changes in individuals^(28)^. Pellegrini *et al*. showed a strong association between weight gain, depression and anxiety during the outbreak^(30)^. During the COVID-19 pandemic in Poland, a total 30 % of the population gained weight^(31)^. In Italy, researchers noted that 48·6 % of the respondents reported weight gain^(32)^. In the present study, similar to Italy, 50·9 % of participants reported weight gain while 32·1 % of those reported weight loss during the epidemic. We also found that the DAS levels of participants whose body weight decreased and increased were significantly higher than participants whose body weight did not change.

Studies have shown that depression, stress and anxiety are higher in obese participants^(33–35)^. Contrary to these studies, in our study, underweight participants had higher DAS levels than normal-weight and overweight participants. Similar to our results, Barcın-Guzeldere *et al*. stated that BMI was negatively correlated with perceived stress scores^(5)^. These findings can be explained by the different responses of subjects to stress^(8)^. The higher perceived stress in individuals with low BMI proposes that COVID-19 stress may suppress appetite^(5)^.

To support the immune system during the COVID-19 pandemic, maintaining a healthy and balanced diet and being physically active are recommended^(36)^. Physical activity appears to play an important role in the links between resilience and depression^(37)^. Ammar *et al.* indicated that there was a noticeable decrease in the prevalence of physical activity during the pandemic, while time spent on sedentary behaviours increased^(38)^. G´ornicka *et al.* revealed that staying at home during the pandemic caused undesirable eating behaviours such as overeating and snacking and reduced the level of physical activity^(39)^. The WHO warned that adults should perform 150 min/week of moderate-to-intensity physical activity to achieve health benefits^(40)^. In the present study, the majority of individuals (73 %) reported that they did not follow the exercise recommendation of at least 150 min/week during the pandemic. The constraints such as limited space and boredom caused by home isolation probably affected individuals’ physical activity levels and their weight. This is important because the practice of physical activity is linked to the individuals’ physical and psychological well-being^(28)^. In our study, in accordance with the literature, it was determined that participants who exercised at least 150 min/week during the outbreak had lower levels of DAS than those who did not. Similar to our results, Maugeri *et al.* reported that the reduction of total physical activity had an extremely negative effect on psychological health^(41)^. According to this evidence, compliance with the physical activity recommendations is a critical strategy for psychological well-being during the pandemic.

It is known that emotional alterations tend to affect the eating behaviours of individuals. These fluctuations can cause an increase or decrease in food intake^(42)^. It has been reported that perceived stress was significantly associated with emotional eating^(43,44)^. Liboredo *et al.* stated that the participants snacked more frequently and increased their food intake during the quarantine period^(44)^. In the study conducted in Türkiye, it was observed that the majority of the participants had changes in their emotional states such as anxiety, stress, and irritability after COVID-19, and these changes increased their food intake^(45)^. In line with the literature, this study showed a positive correlation between negative EMAQ and DAS levels. Moreover, in the current study, it was determined that participants who stated that they skipped the main meal during the outbreak had higher DAS levels, and participants who declared that they had an adequate and balanced diet had lower DAS levels. However, skipping meals was only associated with depression in regression analysis. Similar to our results, Pinchoff *et al*. demonstrated that skipping meal was associated with depressive symptoms during the pandemic^(46)^. These findings can be explained by the loss of appetite which is common in the presence of depression^(8)^.

Overweight people tended to eat more when confronting negative emotions, negative situations, compared with normal-weight and underweight people^(13,47)^. In a study conducted on Turkish adolescents, a positive relationship was found between BMI and emotional eating^(11)^. Similarly, in our study, the eating tendency of overweight and obese people was found to be greater than that of normal-weight and underweight participants in the presence of a negative emotion or situation. In addition, BMI was positively correlated with EMAQ-negative scores. Moreover, it was reported that males demonstrate more eating behaviour than females in a negative mood or situation^(47)^. However, there were no gender differences for negative emotions in our study. The reason for this difference may be geographical and cultural effects. As we all know, a pandemic puts tremendous pressure and stress on our lives and alters almost every aspect of our lifestyle; it is unavoidable that eating habits will be affected, particularly in participants who already overeat. Therefore, obese participants who awareness emotional eating attacks or an increase in food consumption during the pandemic should consult an expert to prevent weight gain.

The present study had several limitations. The study is constrained firstly by its cross-sectional design and lack of longitudinal follow-up. Even though we tried to reach more male individuals, gender distribution was not balanced. We were unable to evaluate the participants’ DAS levels and emotional eating behaviours before the pandemic. The results cannot be attributed to the pandemic per se as their emotional state prior to the pandemic is not known. Body weight, height and all other data were based on self-reported. Therefore, the measure used was not validated, but due to the pandemic, a direct evaluation of these anthropometric measurements was not possible by a researcher. Moreover, several biases inherent in our study should be noted: self-reported bias, self-selection bias and sampling bias as we used a snowball sampling technique. As Türkiye is a vast country and Internet access is still limited in many regions, our results cannot be generalised to the whole population in Türkiye.

The present study had also main strengths. One is that it is the first study to investigate the prevalence and predictors of perceived DAS levels and emotional eating behaviours during the COVID-19 pandemic in the Turkish adult population. This study provides preliminary data that can be used in future studies to determine the DAS levels and emotional eating behaviours during the pandemic. Second strength of this study is its large sample size.

In conclusion, the results of this study showed that approximately one out of every three participants reported moderate to severe perceived DAS during the pandemic. The prevalence of DAS symptoms can be considered high in our study. Perceived DAS was significantly correlated with emotional eating. We thought that the pandemic itself led to the development of negative emotions, and that these negative emotions caused changes in eating habits. In addition, the uncertainty in the period can lead to the consumption of unhealthy foods to regulate negative moods, and this consumption can cause an increase in body weight. The predictors obtained in the current study suggest that healthy diet and lifestyle behaviours are part of psychological well-being and emotional eating behaviours. We hope that this present study will help in the development of strategies through understanding the predictors related to DAS levels and emotional eating behaviours during the pandemic period.
